# Rehabilitation Methods for Patients with Geographic Atrophy due to Age-Related Macular Degeneration and Effects of Rehabilitation on Quality of Life

**DOI:** 10.1155/2023/3389750

**Published:** 2023-07-08

**Authors:** Damla Erginturk Acar, Figen Batioglu, Aysun Idil, Esra Sahli, Dincer Goksuluk

**Affiliations:** ^1^Ankara University Graduate Faculty of Health Sciences, Ankara, Turkey; ^2^University of Health Sciences, Ankara City Hospital, Ankara, Turkey; ^3^Department of Ophthalmology, Ankara University Faculty of Medicine, Ankara, Turkey; ^4^Department of Biostatistics, Erciyes University Faculty of Medicine, Kayseri, Turkey

## Abstract

**Purpose:**

The purpose of the study is to evaluate the low vision rehabilitation methods and to investigate the effect of visual rehabilitation on quality of life in patients with low vision due to geographic atrophy from age-related macular degeneration (ARMD).

**Methods:**

The better-seeing eye of 78 patients with geographic atrophy due to ARMD were included in the study. Sociodemographic characteristics, ophthalmological examination findings, and preferred low vision aids for near and distant were recorded. Fifty-seven patients who preferred to use a low vision aid device in daily life were considered as a rehabilitation group, whereas 21 patients who did not use any device were considered as a control group. The National Eye Institute Visual Function Questionnaire (NEI-VFQ-25) was applied to all patients at the initial examination and at least 6 months after the initial examination.

**Results:**

In the rehabilitation group, statistically significant increases were found in the overall composite score, and general vision, near and distance activities, social functioning, mental health, role difficulties, and dependency subscale scores of the NEI-VFQ-25 quality of life scale after low vision rehabilitation (*p*=0.009 for general vision, *p* < 0.001; for others). In the control group, there was no statistically significant change in any of the subscale scores or the overall score of the scale (*p* > 0.05). All patients (*n* = 78) were recommended to use at least one low vision aid for near vision. Hyperocular glasses were recommended for 77 patients (98.72%), magnifiers for 15 patients (19.23%), electro-optical devices for 2 patients (2.56%), and telemicroscope for one patient (1.28%). Furthermore, 17 patients (21.8%) were prescribed more than one low vision aids. However, for distance vision, only 29 patients (37.18%) received a recommendation for a low vision aid.

**Conclusions:**

Low vision patients with ARMD-related geographic atrophy should meet with low vision aids as soon as possible and should be included in low vision rehabilitation programs.

## 1. Introduction

According to data from the World Health Organization (WHO), 285 million people around the world have low vision problems [1]. Age-related macular degeneration (ARMD) constitutes an important part of the causes that lead to this condition. It is estimated that 300 million people in the USA will have ARMD by 2040 with the increase in life expectancy [2]. Geographic atrophy, also called atrophic ARMD, is an advanced form of ARMD characterized by irreversible atrophy of the retinal pigment epithelium, photoreceptors, and choriocapillaris. Depending on location and size, geographic atrophy causes variable and often significant effects on patients' visual function and vision-related quality of life (VRQoL) [3].

Low vision rehabilitation aims to make patients happy, independent, and productive individuals who are at peace with themselves by contributing to the functional use of their current vision levels [4]. The use of a quality-of-life questionnaire is important to determine the effectiveness of low vision rehabilitation methods. There is no precise definition of success in low vision rehabilitation, but it may be considered “the time the patient finds a device useful and uses it to solve one or more vision problems” [5].

The most desired activities for patients with low vision due to ARMD are near activities such as reading newspapers and books and seeing their cell phones. For this purpose, hyperocular glasses which provide a wide field of view are frequently preferred near vision aids for patients who are new to low vision rehabilitation. Telescopes are the most preferred optical systems for distance vision. Patients may also be prescribed filtered glasses to reduce glare and increase contrast indoors and outdoors, depending on their needs [6].

We aimed to evaluate the low vision rehabilitation methods in patients with ARMD-related geographic atrophy and to evaluate the effect of visual rehabilitation on the quality of life in these patients by using the National Eye Institute visual functioning questionnaire (NEI-VFQ-25) [3].

## 2. Materials and Methods

This study was approved by the Ankara University School of Medicine, Ethics Committee (Registration Number: 2020/I9-561-20). Written approvals were obtained from the patients for all procedures which were carried out in accordance with the Declaration of Helsinki.

Seventy-eight better-seeing eyes of 78 consecutive patients with geographic atrophy due to ARMD who applied to our clinic with the complaint of difficulty in daily tasks related to vision were included in the study. A control group was not formed for our study as it would not be ethically appropriate. However, patients who were prescribed a low vision aid device but did not choose to use it were considered as the control group.

Patients who accepted to participate in our study, who have not received low vision rehabilitation service, and who have not used low vision aid devices before were included in the study. Patients who have ocular pathologies other than ARMD that would reduce their vision, who could not comply with the examination and tests for any reason, and who underwent eye surgery during the study period were excluded from the study.

All participants underwent a detailed ophthalmological examination including the measurement of best-corrected visual acuity (BCVA) for near and distance and anterior and posterior segment examination. Refraction errors of all patients were corrected, distance and near vision aid devices were tried, suitable ones were prescribed, and training programs were organized. The Turkish version of the NEI-VFQ-25 questionnaire [7] was administered to the patients at the initial examination and at least 6 months after the low vision aid was prescribed.

### 2.1. Visual Acuity Measurement

Distance visual acuity was measured with an Early Treatment Diabetic Retinopathy Study (ETDRS) chart at 500 lux illumination from a distance of one or two meters depending on the patient's visual acuity level and recorded in logMAR. When visual acuity was measured from a 1-meter distance, +1.00 Diopter (D) was added in order to prevent the lack of accommodation. If the measurement was made from a distance of 2 meters, the score seen on the chart was divided by 2 and if it was made from a distance of one meter, it was divided by 4.

Near visual acuity was measured under 200 cd/m^2^ luminance illuminations using a reading stand, with Minnesota Low Vision Reading Chart (MNREAD) cards.

### 2.2. Evaluation of Lesion Size and Localization

The size and localization of atrophy were evaluated by the short-wavelength fundus autofluorescence (FAF) images. The lesion size was measured using the “Region Finder” software of FAF imaging semiautomatically [8, 9] in mm^2^ by the same investigator (DEA). The lesion localization was also recorded as subfoveal, juxtafoveal, and extrafoveal.

### 2.3. Quality of Life Scales

The NEI-VFQ-25 questionnaire has two parts, consisting of 25 main questions and 13 additional questions. It was developed to test the psychometric properties of diseases that cause vision loss, evaluate the vision-related quality of life, and evaluate the impact of visual problems on patients' physical, emotional, and social aspects [3]. In the literature, this scale has been shown to have internal consistency and reproducibility and be useful in patients with ARMD-related geographic atrophy [3, 7, 10–12]. In the questionnaire, there are questions about general health, general vision, ocular pain, near and distant activities, visual functioning, mental health, role difficulties, dependency, driving, color vision, and peripheral vision. Driving-related questions were not asked in the questionnaire, since the patients' vision level was not legally sufficient to get a driver's license, and the majority of the patients stated that they had never driven a vehicle in their lives. All scores were calculated by a single investigator (DEA) as detailed before [13].

### 2.4. Application of Low Vision Aid Devices

Low vision aid device trials were performed for all patients for distance and near vision. Hyperocular glasses, magnifiers, telemicroscopes, and electro-optical devices were evaluated for each patient individually, and the proper ones were prescribed for near vision rehabilitation. Galilean and Keplerian telescopes and electro-optical systems were then tested and prescribed for distance vision rehabilitation. In addition, each patient was tested with special filtering glasses for near and distance vision.

The required amount of magnification was calculated with the equivalent viewing distance (EVD) according to the patients' visual acuity, visual field conditions, and binocular vision evaluations. After the patients received the prescribed low vision aid devices, training programs including exercises carried out in the clinic and at home were planned.

### 2.5. Statistical Analysis

Data were analyzed using IBM SPSS version 20.0 software. The numerical data that fit the normal distribution was evaluated using graphical (Q-Q plot, histogram, etc.) and analytical approaches (Shapiro–Wilk's normality test). The mean and standard deviations were used for the normally distributed variables, and the median and quartiles [Q1, Q3] were used for the non-normally distributed variables. Categorical variables were defined using numbers and percentages (%). Wilcoxon signed rank test and paired samples *t*-test were used to compare dependent groups. Student's *t*-test or Mann–Whitney *U* test was used for two groups and Kruskal–Wallis or one-way ANOVA tests were used for three or more groups to compare independent groups. Categorical variables were compared using chi-square tests (Pearson's chi-square, Fisher's exact test, etc.). The relationship between numerical variables was examined using Pearson's or Spearman's correlation coefficient tests. The statistical significance level (*p*) was set as 0.05.

## 3. Results

### 3.1. Sociodemographic Characteristics

Of the 78 patients with low vision due to AMD-related geographic atrophy included in our study, 29 (37.2%) were female and 49 (62.8%) were male. Fifty-seven (73.1%) patients who applied the rehabilitation methods were considered as a rehabilitation group, and 21 (26.9%) patients who did not apply these methods were considered the control group. The mean age of the 78 patients was 75.72 ± 9.44 years (55–94 years). It was 76.44 ± 9.28 years (55–94 years) in the rehabilitation group and 73.76 ± 9.82 years (57–92 years) in the control group. There was no statistically significant difference between the groups in terms of gender and age (*p*=0.247, *p*=0.269, respectively).

When the education levels of the patients in the groups were compared, the education level of the control group was significantly lower than those in the rehabilitation group (*p*=0.016, Table 1).

### 3.2. Ophthalmological Examination and Imaging Findings

The mean BCVA in the better-seeing eye at distance and near were significantly better in the rehabilitation group compared to the control group (0.72 ± 0.26 vs. 0.85 ± 0.22 logMAR, *p*=0.008, 3.22 ± 2.97 vs. 4.30 ± 2.89 M, *p*=0.039, respectively).

The mean area of atrophy was significantly greater in the control group compared to the rehabilitation group (7.78 ± 4.70 mm^2^ vs. 15.46 ± 9.37 mm^2^, *p* < 0.001).

Fifty-two (91.23%) patients had subfoveal, and 5 (8.77%) patients had juxtafoveal atrophy in the rehabilitation group, whereas 20 (95.24%) had subfoveal, and one (4.76%) had juxtafoveal atrophy in the control group. There was no statistically significant difference between the groups in terms of atrophy localization (*p*=1.000).

### 3.3. Quality of Life Questionnaire Scores

The mean overall composite score of the quality of life questionnaire in all participants (*n* = 78) was 46.85 ± 11.58 points (Table 2). When the correlation between the baseline overall composite score of the quality of life questionnaire and demographic characteristics was investigated, there was a statistically significant negative correlation between the overall composite score and age, duration of complaints, and BCVA for near (*p* < 0.001, *r* = −0.1138; *p*=0.015, *r* = −0.0117; *p* < 0.001, *r* = −0.3563, respectively). There was no statistically significant correlation between gender, education level, BCVA for distance, atrophy size, and location. The mean subscale scores of the quality of life questionnaire at the first visit are shown in Table 2.

The baseline median values of the quality of life scales in the rehabilitation and control groups are shown in Table 3. While the general vision and vision-specific social functioning subscale scores in the rehabilitation group were statistically higher than the control group (*p* < 0.001 and *p*=0.011, respectively), there was no statistically significant difference in overall composite and other subscale scores.

The median values of the overall composite and subscale scores of the quality of life scale in the rehabilitation group before and after rehabilitation are shown in Table 4. A statistically significant increase was found in the overall composite score, general vision, near activities, distance activities, and vision-specific variables after rehabilitation (*p*=0.009 for general vision, *p* < 0.001 for others).

The median values of the overall composite and subscale scores of the quality of life scale in the control group at baseline and last visit are shown in Table 5. No statistically significant change was observed in either the overall composite score or the other subscales of the questionnaire. The mean time elapsed between the administration of the two quality of life questionnaires was statistically similar in the rehabilitation group (10.75 ± 1.69 months (7–14 months)) and the control group (10.57 ± 1.66 months (8–14 months)) (*p*=0.670).

### 3.4. Low Vision Aids Used for near and Distance

We determined that all participants (*n* = 78) were recommended at least one low vision aid for near vision. Seventy-seven (98.72%) patients were prescribed hyperocular glasses 77, 15 (19.23%) patients magnifiers, 2 (2.56%) patients electro-optical systems, and one (1.28%) telemicroscope. Also, 17 (21.80%) patients were prescribed more than one low vision aid for near vision. However, we found that only 29 (37.18%) patients have been recommended a low vision aid (telescopic glasses for all of them) for distance vision.

In the rehabilitation group (*n* = 57), all participants were recommended at least one low vision aid for near vision. Hyperocular glasses (the mean 7.89 ± 3.74 D) were prescribed for 56 (98.25%) patients, magnifiers for 15 (26.32%) patients, electro-optical system for 2 (3.51%) patients, and telemicroscope for one (1.75%) patient. Twenty-five (43.86%) patients in the rehabilitation group were recommended one low vision aid (telescopic glasses for all of them) for distance vision. Also, 26 (45.61%) patients preferred glasses with 450 nm filters in addition to the low vision aid devices for distance and near.

In the control group (*n* = 21), all participants were recommended at least one low vision aid for near vision (the mean 8.29 ± 4.19 D hyperocular glasses). Sixteen (76.19%) of them stated that they did not receive the recommended low vision aid because they thought it would not be useful, whereas 5 (23.81%) patients stated that they bought the recommended device but could not use it because they could not adapt. Also, 4 (19.05%) patients did not buy telescopic glasses because they found them too expensive.

## 4. Discussion

In this study, we found a statistically significant increase in the overall composite score, general vision, near and distance activities, vision-specific social functioning, mental health, role difficulties, and dependency subscale scores after the mean 10.75 months of low vision rehabilitation in these patients. No statistically significant change was observed in the overall composite score or subscales of the NEI-VFQ-25, which was repeated after an average of 10.57 months in patients (considered the control group) who did not receive/use the prescribed low vision aid devices.

There are a limited number of studies in the literature in which the NEI-VFQ-25 was applied to patients with ARMD-related geographic atrophy [3, 8, 12, 14, 15]. Ahluwalia et al. applied that questionnaire to 206 patients with central geographic atrophy and 198 patients with exudative ARMD and examined the change in the overall composite score on the questionnaire with the progression of the disease found mean overall composite scores of 73.1 and 75.7 points, respectively, at earlier stages of the disease [14]. They also determined 1.99 and 0.49 point reductions per year in average scores, respectively, whereas these reductions were 1.68 and 3.30 points per year, respectively, with the progression to advanced ARMD.

Patel et al. applied the NEI-VFQ-25 to 137 patients with geographic atrophy and 52 participants without geographic atrophy [12]. The mean of overall composite scores were 53.1 and 84.5 points, respectively, in patients with geographic atrophy and without geographic atrophy. Sivaprasad et al. found a mean overall composite score of 61.7 points in 100 patients with ARMD-related geographic atrophy [3]. We found that the mean overall composite score of our patients was quite low, 46.85 points. In fact, these studies and ours demonstrate the need for the rehabilitation of patients with ARMD.

Künzel et al. performed ophthalmic examinations, including fundus autofluorescence (FOF) measurements and the NEI-VFQ-25, in 87 patients with ARMD-related geographic atrophy [8]. They followed up with the patients for an average of 1 year and monitored changes in the questionnaire scores and the patients' clinical findings. They found a mean overall composite score of 69.96 points, a mean near activities subscale score of 41.67 points, and a mean distance activities subscale score of 58.33 points. These values were 46.85, 25.05, and 34.29 points, respectively, in our study, and 53.1, 25.6, and 26.4 points, respectively, in Patel et al.'s study [12]. We think that the reason for the higher scores in Künzel et al.'s study is the higher BCVA levels (mean 0.3 logMAR) and the smaller atrophy size (mean 7.45 mm^2^) [8]. The mean BCVA level was lower (mean 0.76 logMAR) and the mean atrophy size was greater (mean 9.85 mm^2^) in our study. In the study by Patel et al., the mean BCVA level was 0.6 logMAR, while the atrophy size was not mentioned [12].

In accordance with the literature, our patients got the highest mean scores from the color vision (97.44 points), ocular pain (96.47 points), and peripheral vision (80.13 points) subscales, whereas they got the lowest mean scores from the near activity (25.05 points), general vision (32.47 points), role difficulties (33.29 points), and distance activities (34.29 points) subscales of the NEI-VFQ-25. Pain in and around the eye, color vision problems, and peripheral vision disorders are not expected in ARMD [16, 17]. Due to central scotoma occurring in geographic atrophy, patients often have difficulty in near activities such as reading books and newspapers. There is a strong relationship between reading performance and quality of life, and reading generally constitutes the primary rehabilitation aim of visually impaired individuals [18].

The mean general health subscale score in our study was low (47.60 points), consistent with data in the literature [12, 16, 17, 19]. Considering that the mean age of our patients was 75.72 years, this is an expected result due to accompanying systemic diseases. Indeed, Patel et al. determined the mean general health scores of patients with and without geographic atrophy of 48.0 and 49.0 points, respectively [12].

We found vision-specific social functioning, mental health, and dependency subscale scores of 47.86, 53.55, and 63.06 points, respectively. Patel et al. found scores for the same subscales of 64.7, 53.1, and 62.0 points, respectively, in patients with geographic atrophy and of 98.6, 91.6, and 98.9 points, respectively, in participants without geographic atrophy [12]. These results show that patients with ARMD become psychosocially dependent on the decrease in their visual functions. Visual rehabilitation methods will positively affect not only the patient's visual functions but also their psychosocial status [20]. Caballe-Fontanet et al. detected statistically significant increases in general vision, near and distance activities, ocular pain, color vision, vision-specific mental health, role difficulties, and dependency subscale scores in the NEI-VFQ-25 using only filter glasses in 79 patients with nonexudative ARMD [21]. In our study, 45.6% of the patients in the rehabilitation group preferred the 450 nm filter glasses in addition to low vision aids for near and distance.

Scilley et al. found a strong positive correlation between the BCVA level of the better eyes of patients with ARMD and the subscales of near and distance activities and social function [22]. Künzel et al. found a relationship between the mean overall composite score and the BCVA level of the better eye [8]. We determined a statistically significant negative correlation between the overall composite score and age, duration of complaints, and BCVA level at near in our study.

Similar to our study, Scilley et al. reported that, among their participants, 85 of them used low vision aid devices, whereas 36 did not [22]. They found that patients using a low vision aid device had significantly higher distance activities and vision-specific social functioning subscale scores of the NEI-VFQ-25 than the patients not using one. In our study, the control group had significantly lower education levels, lower BCVA levels at both near and distance, lower general vision and vision-specific social functioning subscale scores of the NEI-VFQ-25, and greater geographic atrophy area. We did not find any statistically significant difference between the two groups in terms of age, sex, duration of complaints, localization of geographic atrophy, or NEI-VFQ-25 scores but found terms of general vision and the vision-specific social functioning subscale. Demirkılınc et al. determined that the low vision aid device use rate was inversely related to patient age, but not to sex, vision levels, or diagnosis of the disease-causing low vision [23]. The authors attributed that outcome to the fact that elderly patients have slowed reactions and limited manipulative skills, and a possible lack of motivation precludes low vision aid device use. Similarly, McIlwaine et al. reported that patients under the age of 65 years were more likely to use a low vision aid device than those over the age of 65 years, although not significantly so [24]. In contrast, we did not find a statistically significant difference in age between the groups that used and did not use low vision aid devices in our study.

Leat et al. reported that patients with better vision are more likely to benefit from low vision aid devices [25, 26]. In our study, the BCVA levels of patients using the low vision aid device were significantly higher than those of the control group, which is consistent with these studies.

In the literature, it has been reported that the rate of low vision aid devices used in patients with low vision ranges from 46% to 80% [5, 23, 24, 27, 28]. In our study, this rate was quite satisfactory, 73%. We think we can increase these rates even more with training during the rehabilitation process by considering the patients' visual acuity levels, age, sociocultural variables, interest in reading, and their expectations of a low vision aid device. In fact, Mitchell and Bradley stated that a better psychological state and motivation in patients were associated with better results during low vision rehabilitation [29].

There are some limitations of our study. First, our participants may not reflect the general population, as they consisted of patients willing to undergo rehabilitation and had the chance to benefit from these opportunities. Second, our study did not have a real control group created by matching the rehabilitation group using demographic and clinical data since it is not ethical to form a control group by not recommending low vision aid devices to patients with low vision who presented to our clinic for rehabilitation. There is no prospective controlled study in the literature investigating the effectiveness of low vision rehabilitation therapy in a homogeneous group of patients with ARMD-related geographic atrophy. In our study, there was a control group consisting of patients who did not use the low vision aid device recommended for them. In this way, the favorable effects of low vision rehabilitation in patients with ARMD-related geographic atrophy were demonstrated in our study. The level of anxiety is high in patients with progressive central vision loss [30]. Using their current functional vision capacity in the best way will enable them to become independent, self-sufficient, productive, self-confident, and happy individuals by enabling them to have social lives and increasing their quality of life.

## 5. Conclusions

Low vision rehabilitation should be added to the treatment protocols of patients with ARMD in addition to multivitamin support and intravitreal injection, and it is important not to ignore their referral for low vision rehabilitation during their current treatment. We think that it is necessary to allocate sufficient time to patients with low vision in retina clinics, to provide education, and motivate them to use low vision aid devices.

## Figures and Tables

**Table 1:** Distribution of educational status of patients in groups.

	Rehabilitation group *n* (%)	Control group *n* (%)	Total	*p* ^*∗*^
Literate	2 (3.5)	2 (9.5)	4	**0.016**
Primary education	22 (38.6)	15 (71.4)	37	
High school	19 (33.3)	4 (19)	23	
Undergraduate	12 (21.1)	0 (0)	12	
Postgraduate	2 (3.5)	0 (0)	2	
Total	57	21	78	

^*∗*^Fisher-Freeman-Halton's exact test. Statistically significant p values are shown in bold.

**Table 2:** The baseline scores of the quality of life scales (NEI-VFQ-25) of all participants.

Quality of life scales	The mean ± SD (min–max)
Overall composite score	46.85 ± 11.58 (22–75)
General health	47.60 ± 12.45 (10–65)
General vision	32.47 ± 10.38 (10–55)
Ocular pain	96.47 ± 8.52 (75–100)
Near activities	25.05 ± 11.13 (4–54)
Distance activities	34.29 ± 14.45 (8–75)
Vision-specific:	
Social functioning	47.86 ± 20.61 (8–92)
Mental health	53.55 ± 14.49 (20–80)
Role difficulties	33.29 ± 17.35 (0–81)
Dependency	63.06 ± 21.37 (13–100)
Color vision	97.44 ± 7.63 (75–100)
Peripheral vision	80.13 ± 21.07 (25–100)

**Table 3:** The baseline scores of the quality of life scales (NEI-VFQ-25) in rehabilitation and control groups.

Quality of life scales	The median [Q1, Q3]		*p* ^*∗*^
	Rehabilitation group (*n* = 57)	Control group (*n* = 21)	
Overall composite score	45.59 [39, 53]	47.50 [38, 52]	0.888
General health	50.00 [38, 60]	45.00 [38, 55]	0.290
General vision	40.00 [30, 45]	30.00 [23, 38]	**<0.001**
Ocular pain	100.00 [100, 100]	100.00 [100, 100]	0.408
Near activities	25.00 [17, 29]	25.00 [15, 33]	0.657
Distance activities	29.17 [21, 50]	33.33 [23, 40]	0.730
Vision-specific:			
Social functioning	50.00 [33, 67]	41.67 [25, 50]	**0.011**
Mental health	50.00 [45, 65]	55.00 [45, 65]	0.973
Role difficulties	25.00 [25, 47]	25.00 [25, 44]	0.804
Dependency	68.75 [50, 78]	68.75 [50, 78]	0.721
Color vision	100.00 [100, 100]	100.00 [100, 100]	0.479
Peripheral vision	75.00 [75, 100]	75.00 [50, 75]	0.613

^*∗*^Mann–Whitney *U* test.

**Table 4:** The scores of the quality of life scales (NEI-VFQ-25) in the rehabilitation group (*n* = 57) before and after rehabilitation.

Quality of life scales	The median [Q1, Q3]		*p* ^*∗*^
	Before rehabilitation	After rehabilitation	
Overall composite score	45.59 [39, 53]	55.88 [48, 62]	**<0.001**
General health	50.00 [38, 60]	47.50 [38, 60]	0.3
General vision	40.00 [30, 45]	45.00 [31, 52]	**0.009**
Ocular pain	100.00 [100, 100]	100.00 [100, 100]	0.317
Near activities	25.00 [17, 29]	37.50 [33, 44]	**<0.001**
Distance activities	29.17 [21, 50]	41.67 [29, 54]	**<0.001**
Vision-specific:			
Social functioning	50.00 [33, 67]	58.33 [46, 75]	**<0.001**
Mental health	50.00 [45, 65]	60.00 [55, 78]	**<0.001**
Role difficulties	25.00 [25, 47]	50.00 [38, 56]	**<0.001**
Dependency	68.75 [50, 78]	75 [56, 81]	**<0.001**
Color vision	100.00 [100, 100]	100.00 [100, 100]	0.317
Peripheral vision	75.00 [75, 100]	75.00 [50, 75]	0.183

^*∗*^Paired samples *T*-test. Statistically significant p values are shown in bold.

**Table 5:** The scores of the quality of life scales (NEI-VFQ-25) in the control group (*n* = 21) at baseline and the second visit.

Quality of life scales	The median [Q1, Q3]		*p* ^*∗*^
	Baseline	Second visit	
Overall composite score	47.50 [38, 52]	46.56 [38, 51]	0.156
General health	45.00 [38, 55]	42.50 [38, 55]	0.429
General vision	30.00 [23, 38]	30.00 [20, 33]	0.061
Ocular pain	100.00 [100, 100]	100.00 [100, 100]	1.000
Near activities	25.00 [15, 33]	25.00 [15, 33]	0.089
Distance activities	33.33 [23, 40]	33.33 [25, 40]	0.483
Vision-specific:			
Social functioning	41.67 [25, 50]	41.67 [25, 50]	0.748
Mental health	55.00 [45, 65]	55.00 [45, 65]	0.546
Role difficulties	25.00 [25, 44]	25.00 [19, 44]	0.059
Dependency	68.75 [50, 78]	68.75 [53, 78]	0.655
Color vision	100.00 [100, 100]	100.00 [100, 100]	1.000
Peripheral vision	75.00 [50, 75]	75.00 [63, 75]	0.317

^*∗*^Paired samples *T*-test.

## Data Availability

Data is available.
